# Higher spatial resolution is not always better: evaluating satellite-sensed sea surface temperature products for a west Pacific coral reef system

**DOI:** 10.1038/s41598-024-84289-0

**Published:** 2025-01-08

**Authors:** Liam Lachs, Simon Donner, Alasdair J. Edwards, Yimnang Golbuu, James Guest

**Affiliations:** 1 School of Natural and Environmental Sciences, Newcastle University, Newcastle upon Tyne, UK; 2 Department of Geography / Institute for Resources Environment and Sustainability, University of British Columbia, Vancouver, BC Canada; 3 Palau International Coral Reef Center, Koror, Palau; 4 The Nature Conservancy, Micronesia and Polynesia, Koror, Palau

**Keywords:** Remote sensing, Coral reefs, Spatial resolution, Mass coral bleaching, Thermal variability, In situ temperature, Climate-change ecology, Marine biology

## Abstract

As marine heatwaves and mass coral bleaching events rise in frequency and severity, there is an increasing need for high-resolution satellite products that accurately predict reef thermal environments over large spatio-temporal scales. Deciding which global sea surface temperature (SST) dataset to use for research or management depends in part on the desired spatial resolution. Here, we evaluate two SST datasets – the lower-resolution CoralTemp v3.1 (0.05° ~ 5 km grid) and the Multiscale Ultra-high Resolution MUR v4.1 (0.01° ~ 1 km grid) – in their ability to predict in situ reef thermal environments (nightly mean and daily maximum) and the severity of past bleaching in Palau, western Pacific Ocean. We expected higher-resolution data to improve prediction accuracy, yet CoralTemp data explained 10% additional variability of in situ temperatures and 70% additional variance in past bleaching. This likely relates to differential data protocols; despite MUR using a higher spatial resolution grid, CoralTemp achieves higher raw satellite observation density in coastal areas by utilising geostationary satellites. MUR SSTs were also consistently more variable. These results reinforce calls to develop more accurate high-resolution SST products for coral reefs. Our paper demonstrates that higher spatial resolution SST data is not necessarily better at predicting in situ thermal environments of coral reefs and local marine heatwave impacts.

## Introduction

Ocean warming and increasingly frequent marine heatwaves are triggering mass coral bleaching and mortality events across the tropics^1,2^. To understand which reefs are naturally less likely to experience marine heatwave stress^3^, and to successfully forecast coral bleaching over large areas^4–6^ requires global remotely sensed temperature datasets of the oceans that are accurate in the vicinity of coral reefs and coastal areas.

Coral reefs are highly heterogenous ecosystems, varying in morphology, community composition and heat stress susceptibility at localised sub-kilometre spatial scales^7–9^. As a result, SST data needs to be at a high enough spatial resolution to accurately capture this heterogeneity. Global gap-free SST products are developed by combining satellite-sensed SST data, in situ temperatures usually from open ocean buoys and drifters, and spatio-temporal interpolation algorithms^10–13^. Further, the accuracy of SST datasets for a given region depend on the local data density (*i.e.* the number of satellite observations per unit area), which can be reduced by cloudy weather (for infrared sensors that require clear skies^12,13^) and in coastal areas (where microwave-estimated SST data are unavailable due to land contamination at certain electromagnetic frequencies^12,14^). Therefore, the performance of different SST datasets can vary regionally depending on their data sources, data handling protocols and interpolation algorithms.

The increase in data storage capacity and numbers of satellite sensors capable of measuring sea surface temperature (SST) at finer (1 km) spatial resolution has allowed a transition from global gap-free products of 50 km resolution to products at ≤ 5 km resolution in recent years^11,13,15^. Temporal resolution of SST products has also increased over time with satellite-derived datasets and climate reanalyses (*i.e.* that combine observations with models) now provided at daily resolution. Such daily data (rather than weekly data which was the best available for some time) is vital for detecting changes in the duration and timing of marine heatwaves and for refining coral bleaching predictions. Sub-daily scale remote-sensed SST data could further advance our understanding of coral response to heat stress, as bleaching and mortality are known to be particularly affected by sub-daily variability, for instance, by extreme high temperatures reached during midday spring low tides^16^. However, capturing sub-daily variability in SST for global gap-free products remains challenging, especially given a lack of sufficient historical data and the confounding effects of solar reflectance on daytime infrared measurements^10,11^.

As climate data products become more widely accessible and are used by a wider array of researchers, there may be a default assumption that higher spatial resolution datasets provide a more accurate and better characterization of the environment. The highest resolution SST data products might be tacitly assumed to provide a best representation of the local effects of marine heatwaves. For instance, the highest resolution global projections of mass coral bleaching conditions statistically downscaled global climate model data using Multiscale Ultrahigh Resolution (MUR) SST v4.1, which is provided on a 0.01° (~ 1 km) grid^5^. This built on other recent work that has relied on lower resolution current SST products, particularly the CoralTemp v3.1 product, provided on a 0.05° (~ 5 km) grid^6,17^. Surprisingly, little work has been done to date to assess the effect of spatial resolution and SST dataset choice on the ability to predict both in situ temperatures for coral reef ecosystems and the local ecological impacts of thermal stress (see assessments using MUR for other ecosystems^18,19^). Here we address this knowledge gap, comparing CoralTemp and MUR with in situ temperature data and bleaching observations, using a case study of Palau in the west Pacific Ocean where extensive historical logger and survey data is available^2,4^. To decouple the influence of spatial resolution and the different data protocols between datasets, we also evaluate a version of MUR re-gridded to CoralTemp resolution.

## Results

**Fig. 1:**
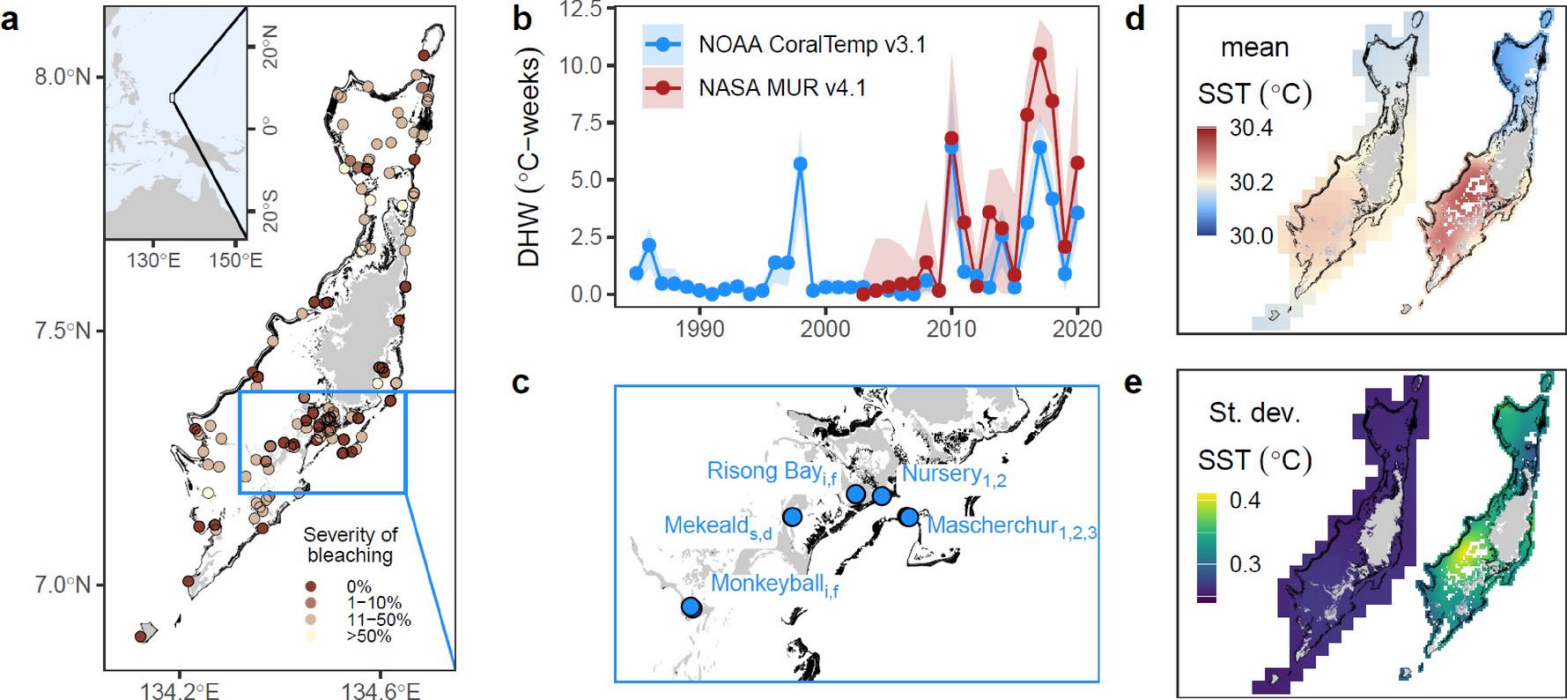
Overview of datasets used in this study of Palau, Micronesia. (**a**) Survey records of coral bleaching since 2003 (points coloured by severity) are shown across Palau, highlighting areas of land (grey), outer reef (black), and the region (blue box, **c**) where in situ temperature loggers were deployed. (**b**) Annual maxima of warm-season accumulated heat stress is shown for CoralTemp v3.1 (blue, since 1998, ~ 5 km grid) and MUR v4.1 (red, since 2003, ~ 1 km grid) as Palau-wide medians (points and lines) and ranges (ribbon). For the 2010 marine heatwave which led to mass coral bleaching and for all SST pixels containing coral reef, the warm-season (June–September) mean SST (**d**) and standard deviation of daily SSTs (**e**) are shown based on the lower resolution CoralTemp v3.1 (left, ~ 5 km grid) and the higher resolution MUR v4.1 (right, ~ 1 km grid). Note the fine scale of mean temperatures (**d**) and temperature variability (**e**).

The Republic of Palau is a small island nation with a range of coral reef habitats, from protected locations in the Rock Islands lagoon to semi-exposed patch reefs in the open lagoon, and an encircling offshore barrier reef (Fig. 1a). The high resolution of MUR clearly resolves kilometre-scale features of the shallow coral reef habitats around Palau compared to the lower resolution 5 km grid cells of CoralTemp which encapsulate a large proportion of open ocean and relatively little reef area (Fig. 1d,e). The annual accumulation of heat stress is more variable and higher on average in MUR than CoralTemp (Fig. 1b). During the 2010 warm season (June–September) which led to mass coral bleaching, seasonal average SSTs recorded for different reefs varied spatially by 30–30.4 °C for MUR compared to 30.1–30.3 °C for CoralTemp (Fig. 1d), and SSTs were more temporally variable for MUR than CoralTemp, with standard deviations of 0.35–0.4 °C and ~ 0.25 °C, respectively (Fig. 1e). Here, we measure SST-derived heat stress in terms of degree heating weeks (DHWs), a metric that captures both the duration and intensity of marine heatwaves, with a standard reference climatology stress temperature used to calculate CoralTemp DHWs and an adjusted climatology used to calculate MUR DHWs. Notably, the approach for calculating non-CoralTemp climatologies (MUR and in situ) and its potential limitations are explained in full detail in the Accumulated Heat Stress section of the Methods. For validation of these SST datasets, we obtained data from an array of calibrated temperature loggers in the central region of Palau across different reef habitat types deployed from 2017 to 2022 (Fig. 1c). Notably, the accuracy of these calibrated loggers was ≤ 0.1 °C (Fig. S1), and instrumental drift was on average < 0.01 °C ± 0.006 °C SD (Fig. S2). We supplement the analysis of in situ temperatures with a test of the relative ability of SST products to predict mass coral bleaching, based on survey data collected since 2003^2,4^ that includes mass bleaching in 2010 and low to no bleaching in other years despite widespread surveys being conducted (Fig. 1a).

### Predicting in situ nightly mean temperatures

**Fig. 2:**
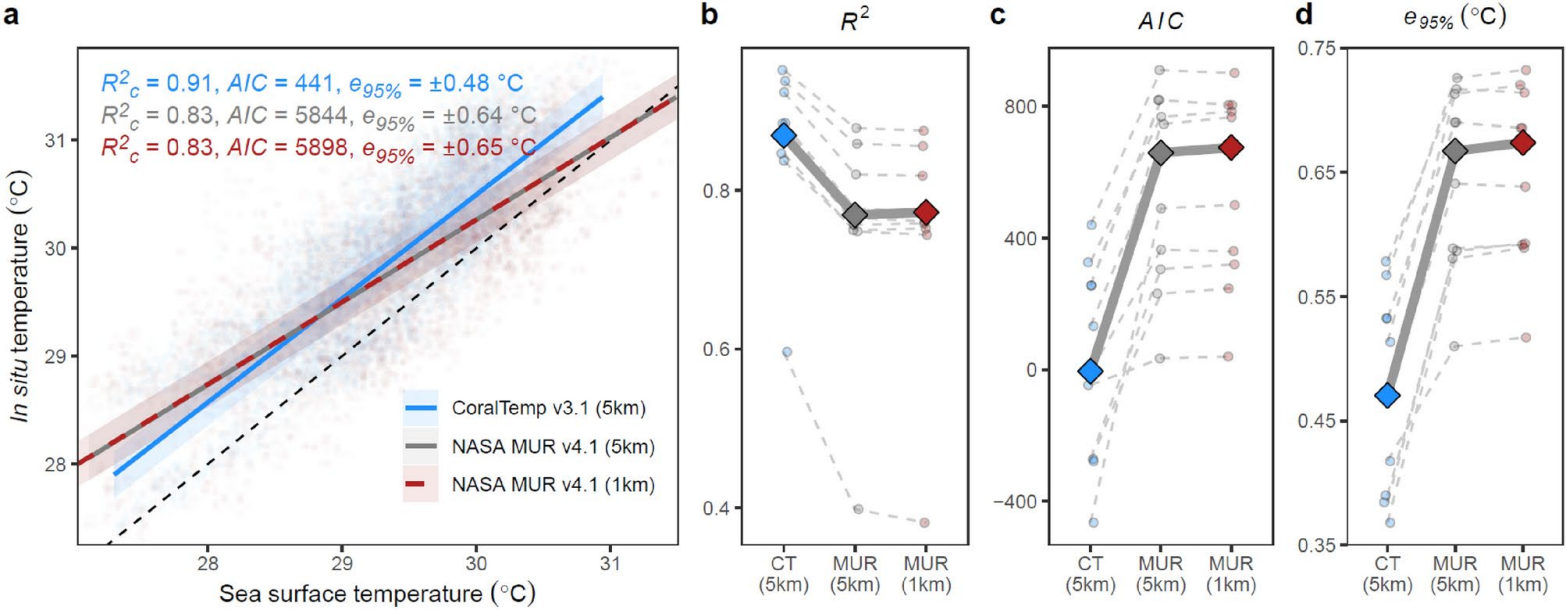
Ability of SST datasets to predict in situ logged temperatures across 11 deployed loggers at 5 sites. To be comparable to SST data, in situ data are first summarised as nightly averages. (**a**) The overall relationship between in situ temperatures and SST from CoralTemp (blue), MUR (red), and MUR re-gridded at CoralTemp resolution (grey), is modelled with a linear mixed effects model, allowing for random intercepts for site and year. Model evaluation metrics show the proportion of in situ temperature variance explained by SST and random effects (conditional *R*^*2*^), overall goodness of fit (Akaike Information Criterion), and model error shown as a 95% error range (*e*_*95%*_, 1.96 × standard deviation of residuals). (**b**–**d**) The same summary statistics shown in (**a**) except based on linear regressions fitted for each site independently (Fig. S3).

Across all sites, CoralTemp explained an additional 8% of in situ variability in nightly mean temperature compared to MUR at native 1 km or at re-gridded 5 km resolution (Fig. 2a). This pattern was characterised by the CoralTemp regression showing the lowest AIC value across all three SST datasets tested. CoralTemp also displayed higher precision, such that error in terms of the e_95%_ metric (*i.e.* the range encapsulating 95% of regression residual values measured as 1.96 × standard deviation of residuals) was ± 0.48 °C for CoralTemp but ± 0.65 °C for MUR. This result remained consistent when comparing the SST products with site-level regressions (Fig. 2b–d, Fig. S3), and when analyses were conducted for the subset of data from the warm season period between June and September when heat stress typically accumulates (Figs. S2, S3).

**Fig. 3:**
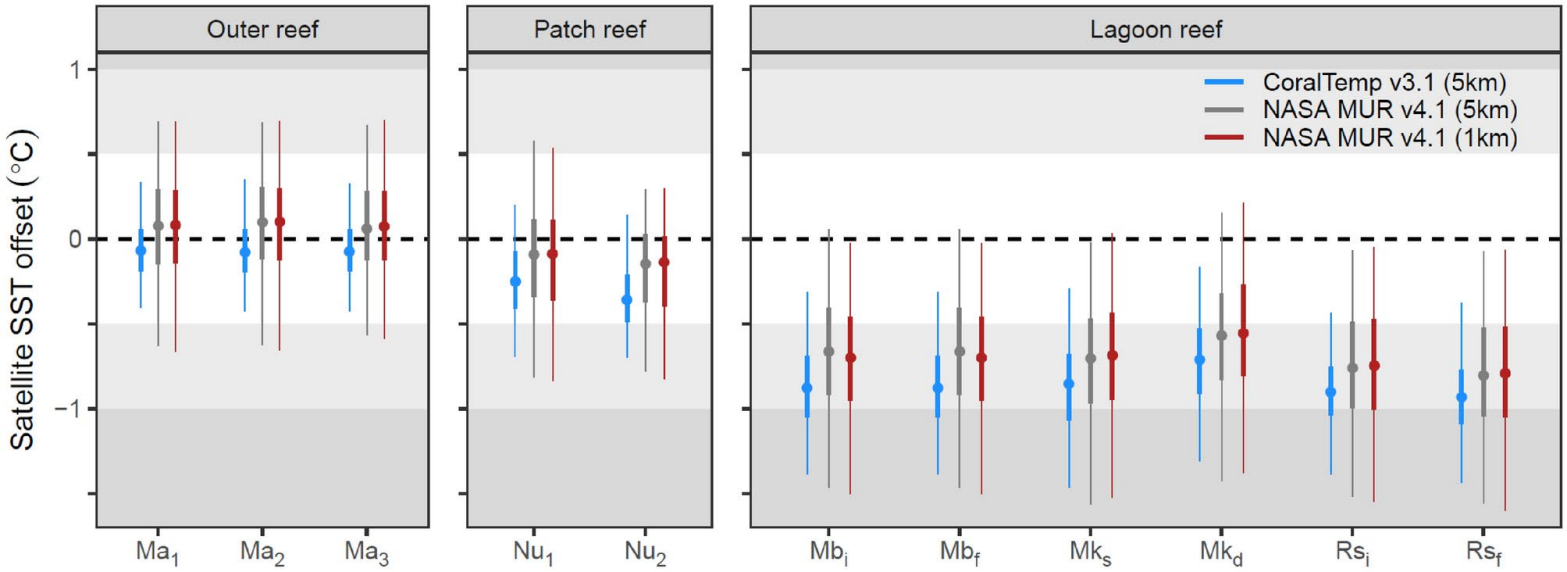
Accuracy and precision of SST datasets by site for CoralTemp (blue), MUR (red), and MUR re-gridded at CoralTemp resolution (grey). A satellite SST offset is the SST value minus the corresponding in situ nightly mean temperature, such that negative values are an underestimation of true temperatures by the satellite. Accuracy can be read here as the median offset (points), while precision can be read as the interquartile range (solid error bar) and the range encapsulating 95% of datapoints (fine errorbar, 2.5^th^ percentile to 97.5^th^ percentile). Site names are abbreviated to Ma (Mascherchur), Nu (coral nursery), Mb (Monkeyball), Mk (Mekeald), and Rs (Risong bay). Corresponding histograms are shown in Fig. S6.

Across sites CoralTemp consistently measured SST at 0.1–0.2 °C lower than MUR (a low level likely within the error margin for SST data and close to the error margin for in situ logger temperatures). The accuracy of these SST data for predicting in situ nightly mean temperatures varied by habitat type, such that both SST datasets achieved accuracy within 0.1 °C for outer reef sites, while accuracy decreased for the patch reef sites (-0.1 to -0.4 °C) and lagoon reef sites (-0.6 to -0.9 °C) that both experience naturally higher day-to-day SST variability (Fig. 3, Fig. S6). Despite the marginally reduced accuracy (~ 0.1–0.2 °C) of CoralTemp for patch reef and lagoon sites, where 5 km grid cells encapsulate both lagoon and open ocean, the precision of CoralTemp was higher across all sites, evident by the narrower range of delta values compared to MUR. This has important implications in the accumulation of heat stress, especially if hot days are over-accentuated by satellite datasets (see below). Provided that satellite bias can be corrected for with in situ data from calibrated loggers, the lower resolution CoralTemp data can provide more accurate estimations of in situ nightly mean temperature stress than MUR for this region.

### Predicting in situ daily maximum temperatures

**Fig. 4:**
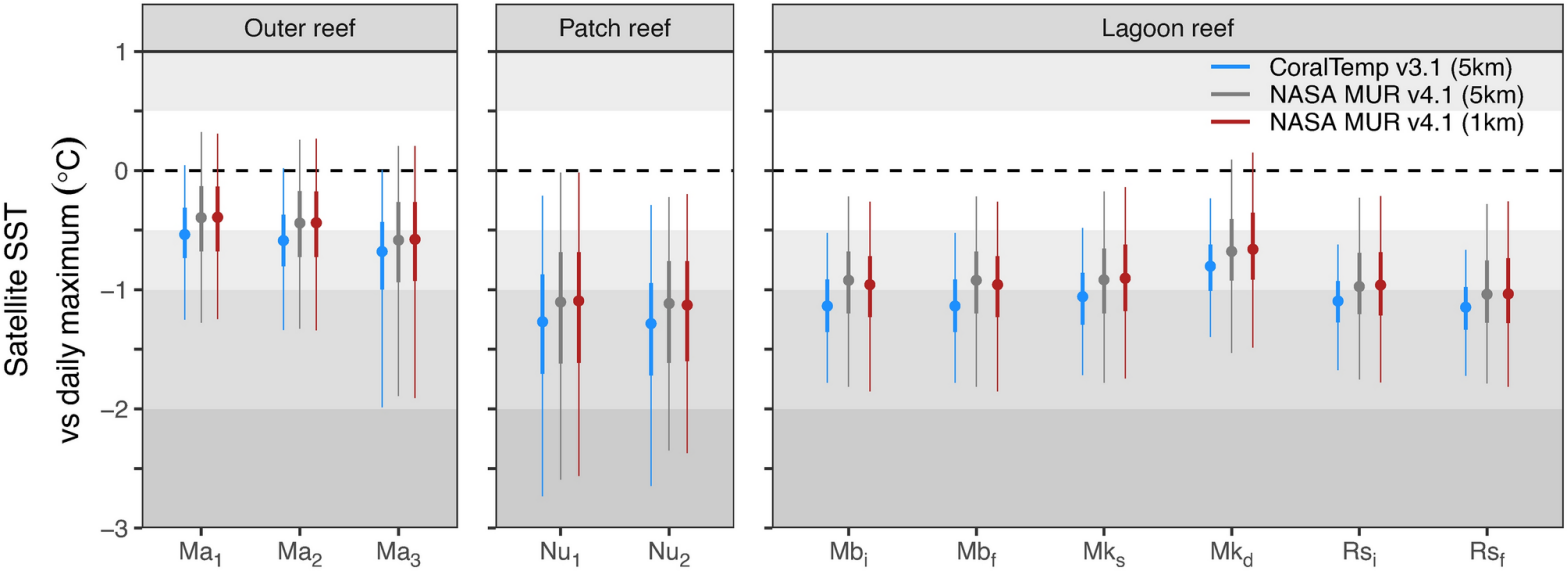
The difference between night-time SST data and in situ daily maximum from hourly logger data by site for CoralTemp (blue), MUR (red), and MUR re-gridded at CoralTemp resolution (grey). The difference is shown as the median (points), while precision can be read as the interquartile range (solid error bar) and the range encapsulating 95% of datapoints (fine errorbar, 2.5th percentile to 97.5th percentile). Site names are abbreviated to Ma (Mascherchur), Nu (coral nursery), Mb (Monkeyball), Mk (Mekeald), and Rs (Risong bay). Corresponding histograms are shown in Fig. S7.

Expectedly, nighttime SST data is consistently lower than in situ daytime peak temperatures (based on hourly logging intervals) by approximately 0.5 °C for the outer reef site, 1.2 °C for the patch reef site, and 1 °C for lagoon reef sites (Fig. 4, Fig. S7). Daytime peaks were recorded as high as 33.6 °C (at the nursery), whereas the maximum nighttime SSTs recorded from pixels encapsulating these sites (over the same period that loggers were deployed) were 30.9 and 31.5 for CoralTemp and MUR, respectively. Despite the fact that MUR SSTs are marginally closer to in situ daily maxima, CoralTemp shows less variability versus daily maxima for the outer reef and lagoon sites, although this pattern is absent from the patch reef site. As such, models for predicting daily thermal maxima using CoralTemp SST rather than MUR SST at the native or 5 km resolution are consistently more accurate and explain more variability in daily maxima (up to an additional ~ 8% of variability, Fig. S8).

### Predicting heat stress and mass coral bleaching

**Fig. 5:**
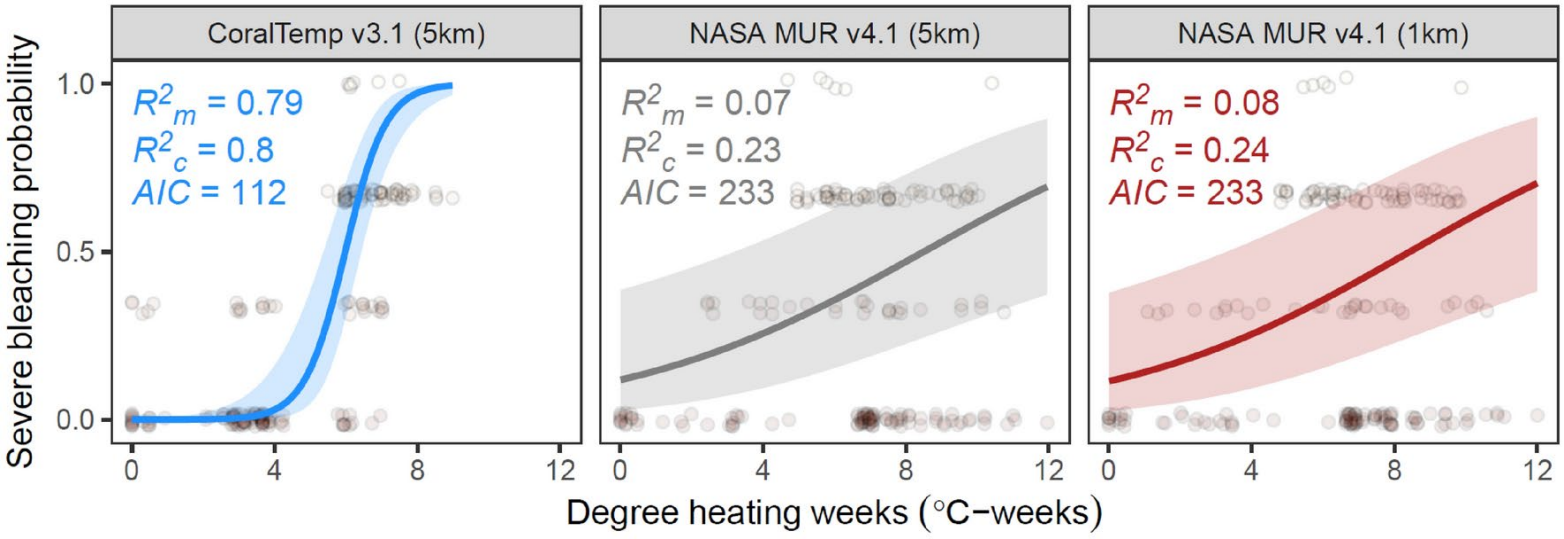
Ability of accumulated heat stress to predict severe bleaching observations (points) in Palau for CoralTemp (blue), MUR (red), and MUR re-gridded at CoralTemp resolution (grey). Heat stress is computed in terms of maximum degree heating weeks. Predicted relationships (lines) from binomial generalised linear mixed effect models (in the form: bleaching ~ DHW) account for latitudinal spatial autocorrelation (for sensitivity analyses refer to^4^) and are shown with 95% confidence intervals (ribbons). Model fit is shown as the proportion of bleaching severity variability explained by DHW alone (marginal theoretical R^2^), the proportion explained by the combination of DHW and spatial autocorrelation (conditional theoretical R^2^), and the Akaike Information Criterion (AIC). Poorer performance of MUR than CoralTemp is strongly driven by the considerable overestimation of DHW by MUR compared to CoralTemp in 2016 and 2017 (Fig. 1b) when almost no bleaching was recorded during surveys.

For coral bleaching data collected in Palau since 2003, the probability of severe bleaching (i.e. > 50% of corals bleached) is strongly predicted by the accumulation of heat stress from the CoralTemp SST data (as shown from an analysis of the full bleaching dataset from 1998 onwards^20^). CoralTemp DHWs alone predicted 79% of variation in bleaching severity (Fig. 5). However, MUR DHWs had a very low capacity for predicting variation in bleaching severity (< 10% of variability explained), with a stronger predictive ability from the spatial autocorrelation random effect (an additional 16–17% variance explained by latitudinal group). The improved accuracy of CoralTemp DHW models compared to MUR DHW models was evident with lower AIC values. More accurate bleaching predictions achieved by CoralTemp than MUR were driven by the differential detection of heat stress in 2016 and 2017 between the two SST datasets. In these two years, underwater surveys reported almost no incidences of bleaching, yet MUR detected extreme DHWs (majority of data > 9 °C-weeks) that far exceeded the levels in 2010 that led to mass bleaching (Fig. 1b, Fig. 5 cluster of no-bleaching points on the bottom right of the plot).

**Fig. 6:**
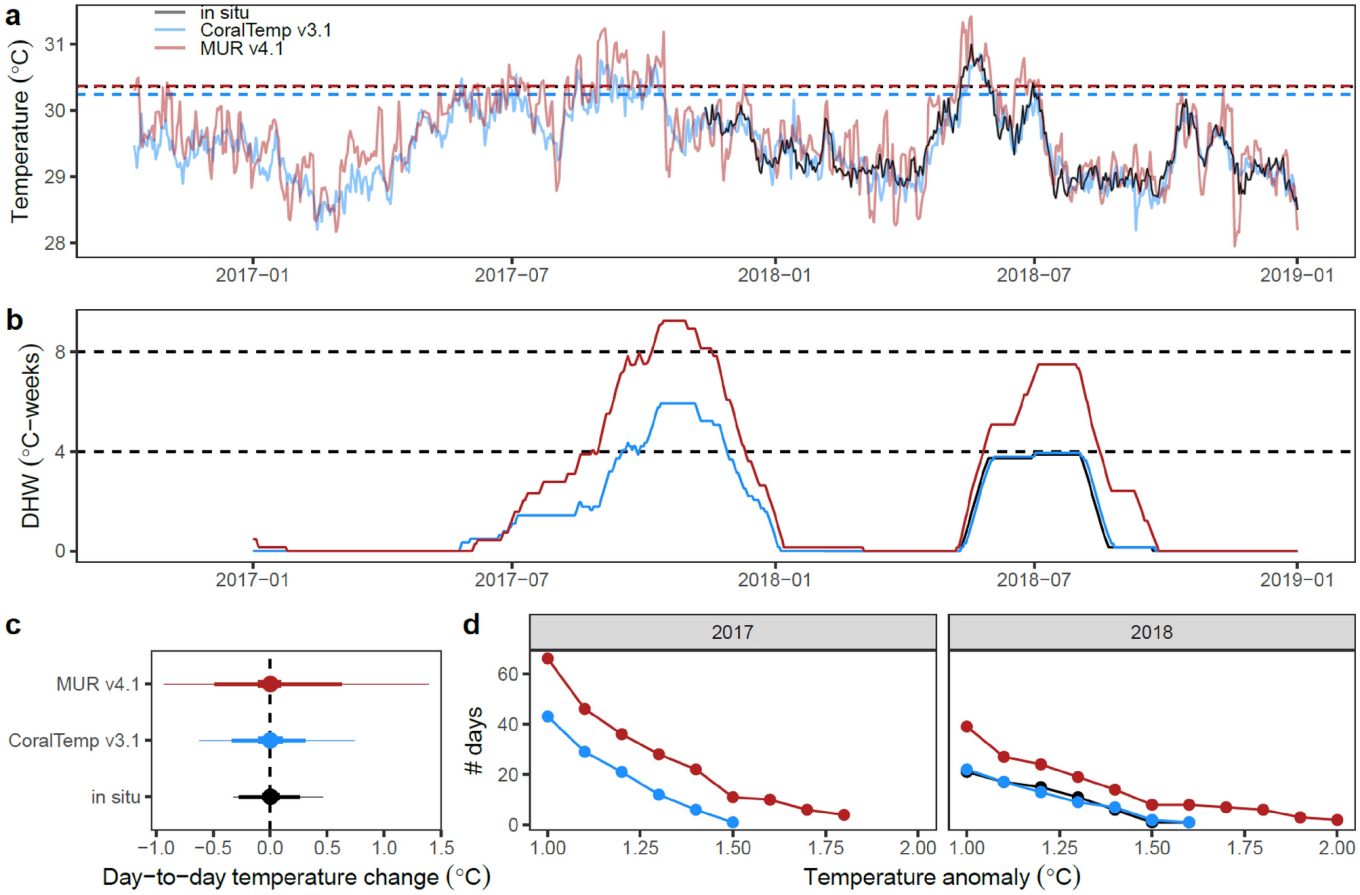
Predictions of heat stress at the outer reef site and day-to-day temperature variabilty. (**a**) Time series of temperature for the pixel encapsulating Mascherchur reef for the 2017 and 2018 marine heatwaves, and in situ temperatures for the 2018 heatwave (shown as the mean across the three loggers at this reef). Dashed lines show the heat stress accumulation threshold (maximum of monthly means – MMM + 1 °C for CoralTemp, and the adjusted MMM + 1 °C for both MUR and in situ data). (**b**) Time series of accumulated heat stress, with close match between CoralTemp and in situ data. (**c**) Distributions of day-to-day changes in temperature, showing the min to max range (fine feather), the 95% range (2.5^th^ percentile to 97.5^th^ percentile, medium feather), the interquartile range (bold feather), and the mean (point). (**d**) Frequency of days with temperatures exceeding different levels relative to the dataset-specific MMM, showing a higher frequency of hot days for the MUR dataset.

While we did not have in situ temperature data for the 2017 marine heatwave—which was the year that drove the low accuracy of bleaching prediction for MUR—a heatwave of similar magnitude occurred in 2018 when we did have in situ temperature data. For Mascherchur reef, in situ temperature and DHWs were closely aligned to those of CoralTemp, reaching 4 °C-weeks, while MUR suggested a higher accumulation of heat stress of almost 8 °C-weeks (Fig. 6b). This is attributable to higher day-to-day (interdaily) variability in the MUR dataset, which is three times that of the in-situ loggers, and almost twice that of CoralTemp; day-to-day changes in temperature vary from -0.9 °C to + 1.4 °C in MUR, -0.3 to + 0.5 °C in the in-situ data, and -0.6 to + 0.7 °C in CoralTemp (Fig. 6c). This leads to MUR featuring more frequent heat stress days which contribute to stress accumulation (Fig. 6d). Since the accumulation of heat stress on a given day is based on all daily SST values exceeding the MMM + 1 °C over the past 84 days, days with spurious overly hot satellite-based SSTs (> MMM + 1 °C) will lead to inaccurate higher levels of accumulated heat stress. This happens more frequently in the MUR data for the study sites, even though the MMM baseline used for computing heat stress is 0.1 °C higher in MUR than CoralTemp (Figs. 3, 6a).

## Discussion

Coral reefs are geomorphological structures that are highly variable in space, driving complex oceanographic dynamics and microclimatic variability^9,21^. Given the continued progression of ocean warming and the susceptibility of reef-building corals to heat stress, there is a growing need for research and management to have access to high spatial resolution information on thermal variability across reefs. CoralTemp and MUR are two satellite SST datasets commonly used to study coral reefs, with the most striking difference between them being the order of magnitude difference in spatial resolution, such that 25 MUR grid cells fit within a single CoralTemp grid cell. Despite this, we demonstrate that in a Pacific island chain with extensive in situ logger data, MUR SST data provides less accurate predictions of in situ nightly mean and daily maximum temperatures. Further, while the warm season accumulation of heat stress based on CoralTemp data explains almost 80% of past records of bleaching severity, MUR-derived heat stress data explains < 10% of variation. Together, our study shows that higher spatial resolution SST data is not necessarily better at predicting in situ thermal environments and ecological impacts of marine heatwaves.

It is likely that one of the major drivers of differences in measured SSTs between MUR and CoralTemp for our study area are their contrasting data sources and data handling algorithms. Microwave SST estimations – which are confounded within 60-100 km of coastlines and so excluded from SST datasets^12^ – are a major data source for MUR^11^ but not used in CoralTemp^10^. In contrast, infrared SST measurements from geostationary satellites – which provide up to 96 observations of the same geographic location per day^13^ – are an input to CoralTemp^10^ but not MUR^11^. While infrared SST measurements from polar orbiting satellites have extremely high global coverage and are used for both CoralTemp and MUR, this data source provides few repeat observations of individual geographic locations per night and so does not contribute to data density as significantly as geostationary satellites^13^. Therefore, for coral reef ecosystems, which are often located near coastal areas, MUR relies on fewer observations per unit area, derived from polar orbiting satellites. In comparison, CoralTemp utilises a much larger density of satellite SST observations both from polar orbiting and geostationary satellites. As such, MUR likely has lower raw data density in coastal areas and is more reliant on interpolation from oceanic observations than CoralTemp. This likely explains (1) the consistently higher SST variability recorded by MUR relative to CoralTemp reported here and in other studies^19^, (2) the higher prediction skill from CoralTemp with regard to in situ nightly mean and daily maximum temperatures, and (3) the considerably more accurate DHW-based predictions of past bleaching severity from CoralTemp rather than MUR. Notably, a larger true day-to-day variability could be expected within smaller spatial areas (although this pattern was still present when MUR 1 km was re-gridded to 5 km), due to factors including tidal excursions, shifting currents, and variable wind and mixing patterns. These factors are more likely to be averaged out across the larger 5 km pixels, whereas tidal excursions or shifting currents would more frequently move surface waters of various temperatures in and out of the smaller 1 km pixel areas. As such, an increased spatial resolution of satellite datasets may require a corresponding increased temporal resolution in order to overcome these variable effects.

Shallow marine ecosystems are under extreme pressure from climate change^22^, as many habitat-forming species are unable to withstand the physiological stress associated to intense marine heatwaves^23–25^. Yet there are still many ecological phenomena that remain uncertain, such as the ability of organisms to acclimatise to warming conditions^26,27^, and the potential for adaptation to warming through natural selection^28^. Further information on fine-scale spatial temperature variability is required to understand how heat stress responses vary with respect to microclimatic variability across habitats^29^, and more detailed spatial data on sub-daily thermal variability is required to understand the effects of nighttime reprieve and daily thermal peaks on organismal responses to marine heatwaves^30,31^. While such data can be gathered from arrays of calibrated in situ temperature loggers, this approach to tracking temperatures is likely to be challenging, costly, and potentially infeasible at the large spatial scales > 100km^2^ at which marine ecosystem management typically takes place^32^, especially for lower income countries that are typically most reliant on coastal marine ecosystems and most at threat from climate change^33^. Therefore, our study reinforces the calls to continue improving the accuracy and spatial–temporal resolution of remote-sensed temperature products developed specifically for coastal marine ecosystems. Further validation and investigation of new remote-sensing developments such as hourly SST datasets^34^ could yield important insights into ecological responses to sub-daily thermal variability.

Ideally, managers and researchers trying to understand thermal variability and the socioecological risks associated to marine heatwaves should aim to ground-truth their temperature observations with data collected from in situ calibrated temperature loggers. Clearly, given the calibration data we show, even high precision loggers can have consistent yet strong offsets. Therefore, it is imperative to calibrate loggers against known standards. Even relatively short time series of in situ temperatures of up to one year can be sufficient to correct historic satellite SST data for locations of interest, and even reconstruct sub-daily thermal variability (*e.g.* midday peak temperature exposures) from the SST data. In the absence of in situ temperature data, SST data still provides a useful first estimate of thermal variability.

Given the spatial heterogeneity of coral reefs and other shallow marine ecosystems, it seems logical to use the highest spatial resolution SST data available in order to support research and management of marine ecosystems. However, our study demonstrates that, at least for our study location, more accurate predictions of in situ thermal environments and marine heatwave impacts can be achieved through using a lower-resolution SST dataset (5 km grid CoralTemp v3.1) designed for coastal research rather than an ultra-high-resolution SST dataset (1 km grid MUR v4.1). This work highlights the need for multi-disciplinary researchers to understand how remote sensed data products are created and which available products can be applied to specific research questions.

## Methods

We compiled SST data from CoralTemp v3.1, a 0.05° × 0.05° latitude–longitude resolution (~ 5 km) dataset available starting in 1985 from the National Oceanic and Atmospheric Administration (NOAA), and MUR (Multiscale Ultrahigh Resolution) v4.1, a 0.01° × 0.01° latitude–longitude resolution (~ 1 km) dataset available starting in 2003 from the Group for High Resolution Sea Surface Temperature (GHRSST). Both datasets are currently developed using nighttime-only observations to reduce the effects of solar radiation on ocean skin temperatures. To assess the ability of SSTs to predict in situ temperatures, we deployed a set of calibrated HOBO WaterTemp Pro v2 temperature loggers set with an hourly recording interval from 2017 to 2022 at 11 fixed locations across five sites and three shallow coral reef habitats: three sheltered lagoon reefs, one patch reef, and one outer reef (for coordinates, deployment time, and depth, see Table S1). Logger locations were all 3–5 m depth, except one logger deployed at 9 m depth (Mekeald_deep_). To assess the ability of SST-derived heat stress to predict bleaching we prepared a 2003-onward subset of previously published mass coral bleaching survey records from Palau. To decouple the influence of spatial resolution and differential data protocols between CoralTemp and MUR, we also evaluate a version of MUR re-gridded to CoralTemp resolution.

### Calibrating temperature loggers

Temperature loggers were calibrated with a certified digital thermometer (TR-1050, ± 0.005 °C accuracy) in 2018 (across two calibrations, half of the loggers in each) and a subset of loggers not deployed in the field were re-calibrated in 2019 to assess instrumental drift. Across each calibration, the following general protocol was followed. Logging at 1-min intervals, the HOBO loggers and TR-1050 were placed in a shared water bath that was pre-cooled to ambient temperature in an air-conditioned room (~ 27–29 °C). The water bath temperature was then increased by a ~ 0.5 °C increment and left for 20 min for temperatures to become stable. This incremental increase-and-wait pattern was repeated until temperatures reached above those likely to be experienced in the field (~ 35 °C). Then, the mean offset of each logger was recorded and subtracted from all data points from that logger. For loggers with multiple calibration the mean was used, given the very low drift observed (0.0075 °C on average).

### Predicting in situ water temperature

To match the same recording window as the nighttime SST data, we calculated nightly averages from in situ temperature data, as well as daily maximum temperatures. Nightly averages were computed for each date from 6 pm the evening before to 6am on the date in question. The ability of SST to predict in situ logged water temperatures was tested independently for both in situ nightly mean and daily maximum temperature, and for each of the SST datasets (CoralTemp, MUR, and MUR_5km_) using a three-pronged approach. Firstly, we fitted an overall model (per SST dataset) testing the effect of SST on in situ temperature using a linear mixed effect model, accounting for among-site and among-year variability with random intercepts for site and year. Secondly, we fitted individual linear regressions for each site independently. Thirdly, we computed the SST offset (SST minus in situ temperature) and plotted the median and distributions to give a measure of accuracy and precision. Model comparisons were conducted based on the Akaike Information Criterion (AIC) where lower values are given for models with a better fit, R^2^ values, and variability in model residuals as a measure of error, computed with the e_95%_ metric (*i.e.* the range encapsulating 95% of regression residual values measured as 1.96 × standard deviation of residuals). As stated previously, this approach was repeated both for the in situ nightly mean temperature (which is directly comparable to the satellite datasets) and for in situ daily maximum temperatures (which SSTs are expected to underpredict, but the precision of these regressions can be used to evaluate the potential of the SST datasets for predicting daily maxima when in situ data is not present). Further, since temperatures during the warm season drive the most significant ecological impacts on coral reefs, there is additional value to optimising predictions during this period of the year. As such, we also repeated these analyses on the subset of data from the warm season only (June–September).

### Accumulated heat stress

Mass coral bleaching is well known to be triggered by intense marine heatwaves and can be predicted successfully given the amount of heat stress that accumulates over the heatwave^30,35^. Here we measure heat stress in terms of degree heating weeks (DHW), following the standard NOAA methodology^10^, as it is the most common measure of accumulated heat stress used in the literature^5,6,30,35^. In brief, DHW on a given day is computed as the sum of the last 12 weeks (84 days) of daily temperature anomalies (HotSpots) relative to a standard local climatological baseline (MMM_CoralTemp_ – maximum of monthly means climatology, see ^36^). Only Hotspots > 1 °C are accumulated, and the result is divided by 7 to achieve a weekly DHW metric, such that values exceeding 8 °C-weeks have been found to be associated with mass coral bleaching. Notably, the MMM_CoralTemp_ is centred on 1988, the midpoint of the baseline period 1985–1993 (excluding 1991–1992 due to volcanic activity in those years, and actually based on data from 1985 to 2012, see Ref.^36^ for details), yet the MUR dataset only begins in 2003. Therefore, for MUR we employed the standard DHW algorithm^10^ with an adjustment of the MMM based on the MMM_CoralTemp_. The MUR baseline (MMM_MUR_) was computed by first calculating the difference in recent MMM (2003 to 2020) between MUR and CoralTemp (ΔMMM_2003-2020_), and then subtracting this value from the standard MMM_CoralTemp_ (MMM_MUR_ = MMM_CoralTemp_ – ΔMMM_2003-2020_). This approach was also used to calculate DHWs for MUR re-gridded at 5 km resolution. We further explore SSTs and in situ temperatures for an outer reef site for 2017–2018. During this period there were two heatwaves, a known lack of bleaching in 2017 (given survey data) and likely also in 2018 (no anecdotal mass beaching reports), and availability of in situ temperature data in 2018. Notably, for in situ DHWs we also adjusted the MMM following the same approach.

The MMM_2003-2020_ calculation we performed does not include the method employed by the Coral Reef Watch of adjusting a PathFinder-based 1985–2012 MMM by the linear regression of monthly means for that time period to centre the resulting MMM on 1988^36^. This is because the pre-2003 data are not available for MUR precluding such an approach. In addition, the CoralTemp SST blends three SST datasets, transitioning in 2002 from (A) the OSTIA SST dataset to (B) the Reprocessed Geo-Polar Blended NESDIS dataset, and then in 2016 from dataset B to (C)the Near-Real Time Geo-Polar Blended dataset. As such, our method assumes that a difference between MUR and CoralTemp SST climatologies during 2003–2020 (SST data B and C), would hold the same trend back in time to 1988 (SST dataset A) if it were possible to estimate differences between MUR and CoralTemp in that period. The MMM_CoralTemp_ uses SST datasets A and B (centred in 1988 so more bound to A), while the CoralTemp MMM_2003-2020_ uses SST datasets B and C. Therefore, there is a possibility that this can introduce some unquantifiable uncertainty on the comparability of the adjusted MMMs (e.g. MUR and in situ).

### Predicting coral bleaching

Underwater coral bleaching survey data used in this study were collected between 2003 and 2017, encompassing the 2010 mass bleaching event^2,4,37^. This dataset is publicly available^2,37^ and provides the timing and coordinates of each survey, as well as a bleaching severity score (based on the percentage of corals bleached) with values of 0 (< 1% bleaching), 1 (1–10% corals bleached), 2 (11–50% corals bleached), or 3 (> 50% corals bleached). We matched each bleaching survey record based on their coordinates and timing to the corresponding annual maximum DHW value recorded in the encapsulating satellite grid cell. The ability of each SST dataset to predict bleaching severity was modelled as a function of annual maximum DHW using generalised linear mixed models (in the form: bleaching severity ~ DHW). Following a previously validated approach for this dataset^4^, severity scores were transformed to a 0–1 variable by dividing the score by 3 and modelled using a binomial response distribution, spatial-correlated uncertainty was accounted for using a three-level latitudinal grouping factor (< 7.3°, 7.3–7.6°, and > 7.6°). Notably, previous work on this Palauan bleaching data with DHWs derived specifically from CoralTemp have shown that modelling bleaching severity as a function of DHW is robust to different error distributions (Gaussian, binomial, beta), different model types (e.g. ordinal regression on untransformed data), and unbalanced designs (e.g. by testing datasets with even numbers of observations per year). We chose the annual maximum DHW metric because some bleaching data (particularly in 2010) did not provide exact dates, but the month and year instead. Therefore, we reran the subsequent analyses using DHW on specific dates instead and assuming the middle day of the month (day 15) for records that were missing a day of month. These analyses yielded similar results, with slightly lower predictive ability for MUR (Fig. S9).

## Supplementary Information

Supplementary Information.

## Data Availability

In situ temperature logger data and all original code (R version 4.0.2) have been deposited at https://doi.org/10.25405/data.ncl.26969983. All datasets analysed are publicly available as of the date of publication. Any additional information required to reanalyse the data reported in this paper is available from the lead author Liam Lachs upon request.
