# Supplementary material for: Higher spatial resolution is not always better: evaluating satellite-sensed sea surface temperature products for a west Pacific coral reef system
Source: Sci Rep. 2025 Jan 8;15:1321. doi: 10.1038/s41598-024-84289-0 (PMC11711397; doi:10.1038/s41598-024-84289-0)
Supplement: Supplementary file 1 — Supplementary Information. [file 41598_2024_84289_MOESM1_ESM.docx]

**Higher spatial resolution is not always better: evaluating satellite-sensed sea surface temperature products for a west Pacific coral reef system**

Liam Lachs^1,2^*, Simon Donner^2^, Alasdair J. Edwards^1^, Yimnang Golbuu^3,4^, James Guest^1^

^1^ School of Natural and Environmental Sciences, Newcastle University, Newcastle upon Tyne, UK
^2^ Department of Geography / Institute for Resources Environment and Sustainability, University of British Columbia, Vancouver, British Columbia, Canada
^3^ Palau International Coral Reef Center, Koror, Palau
^4^ The Nature Conservancy, Micronesia and Polynesia, Koror, Palau

* Corresponding author: liamlachs@gmail.com

**Supplementary Information**


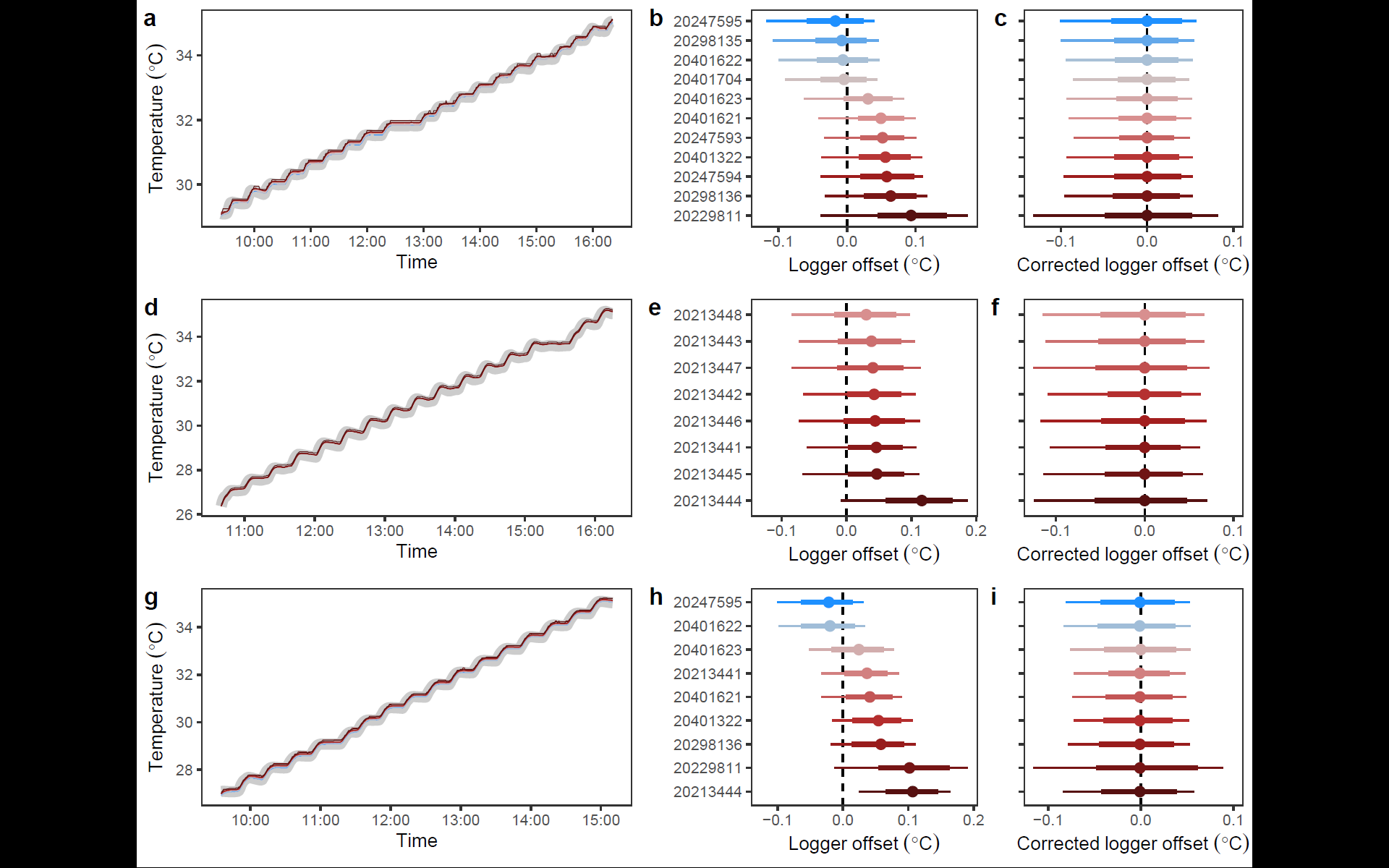


**Figure S1.** HOBO WaterTemp Pro v2 logger calibration and accuracy from three calibrations performed in August 2018 (**a**-**c**), September 2018 (**d**-**f**), May 2019 (**g**-**i**). (**a**,**d**,**g**) Calibration time series of temperature measured in water bath warmed at 0.5°C increments from the certified digital TR-1050 thermometer (broad grey line) and the raw HOBO loggers (fine coloured lines, ID codes as in b, e, and h). (**b**,**e**,**h**) Logger offset (HOBO logger minus TR-1050) for each HOBO logger (y axis) coloured by offset. (**c**,**f**,**i**) HOBO logger precision (Corrected HOBO logger minus TR-1050) is within ±0.1°C. Error bar panels show the mean offset (point) the interquartile range (bold feather) and the 95% range (2.5^th^ to 97.5^th^ percentiles, fine feather).


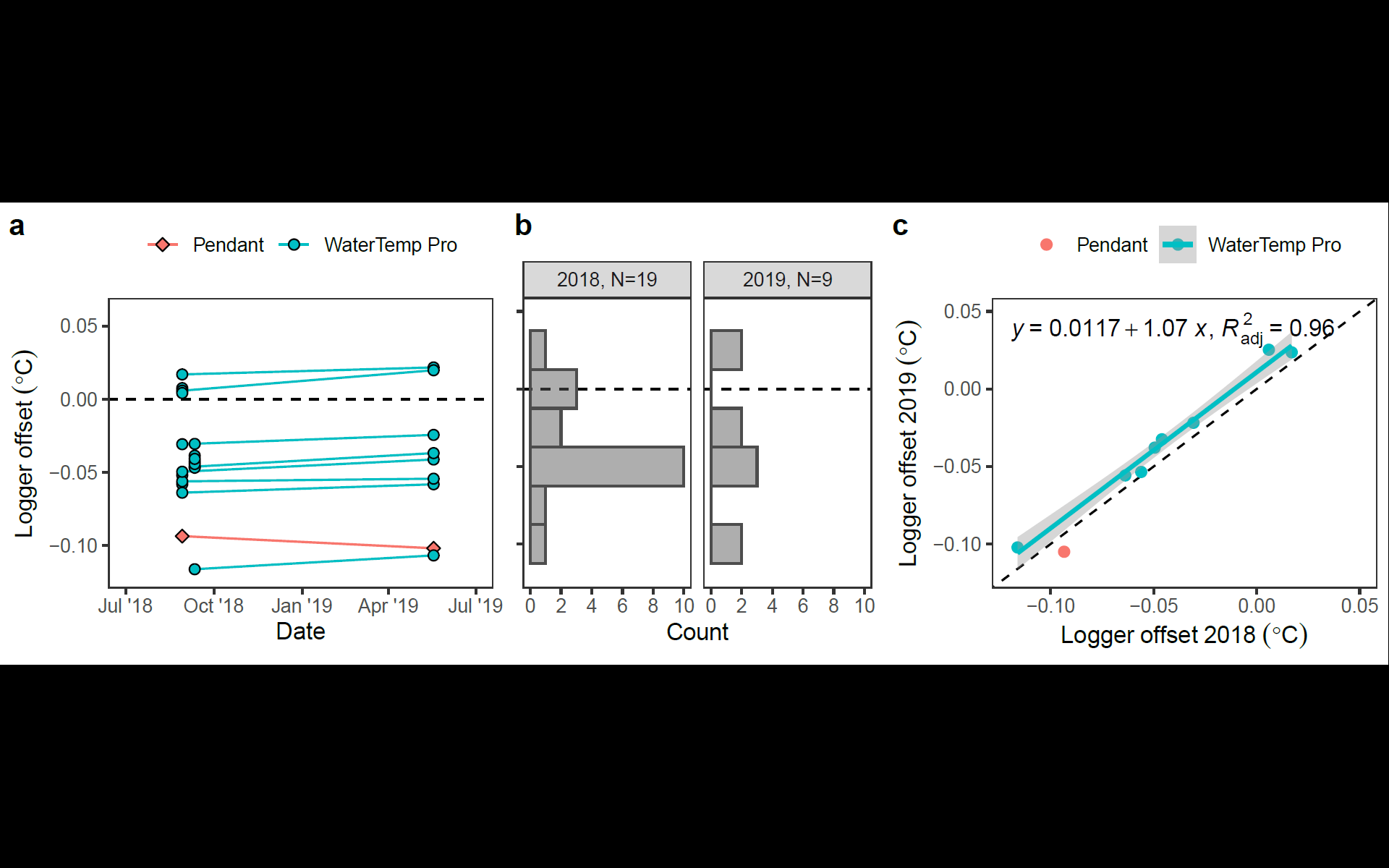


**Figure S2.** Temporal consistency of HOBO WaterTemp Pro v2 logger bias. (**a**) Calibration offsets for individual loggers deployed in this study in 2018 and in 2019. (**b**) Histograms of thermal offsets between years. (**c**) Correlation between 2018 offset and 2019 offset (corrected for each logger to be show annual drift assuming it is consistent, rather than 8-months later drift).


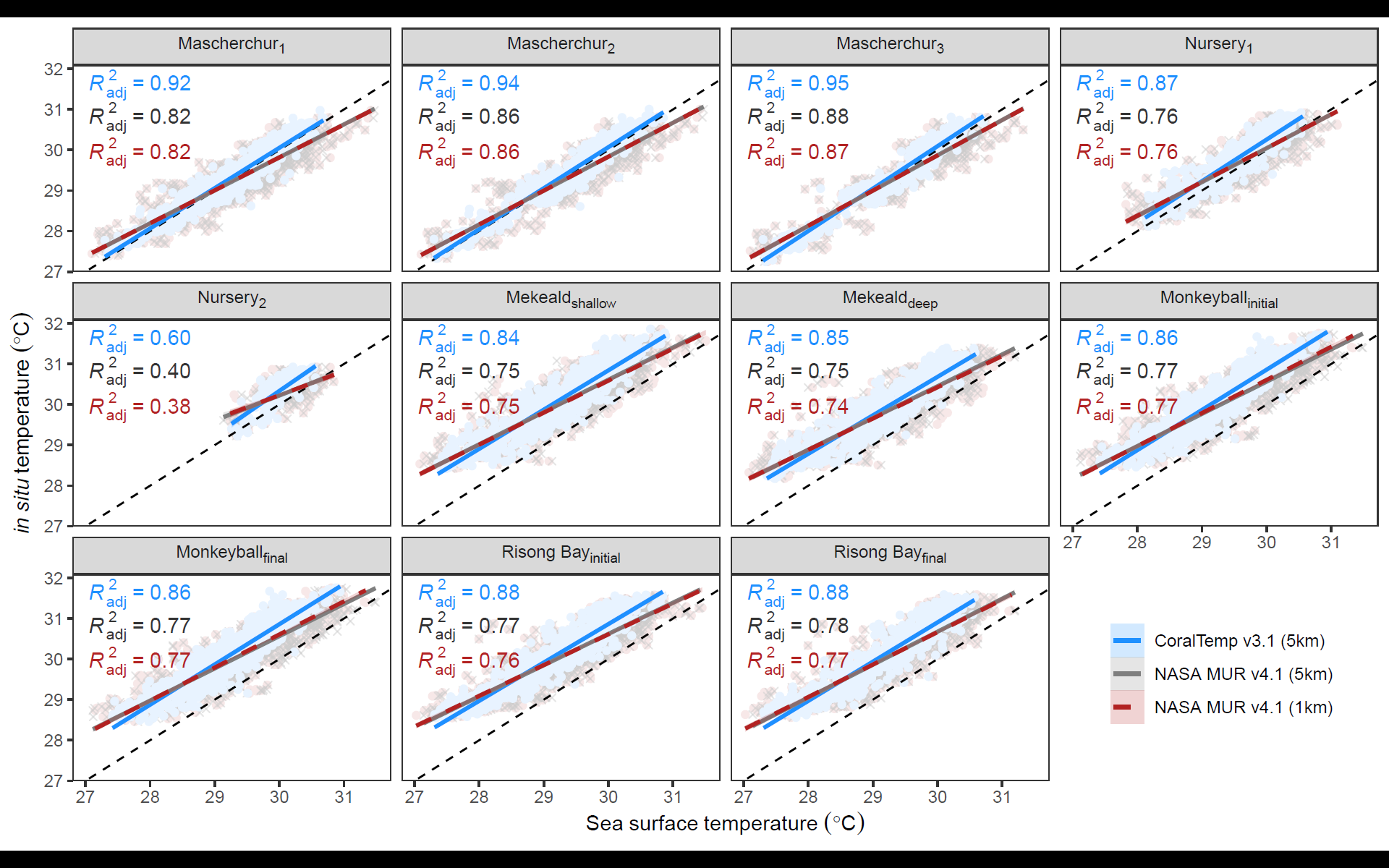


**Figure S3.** Site-specific linear regressions between sea surface temperature (SST) and nightly average in situ logged temperatures supporting Fig. 2b-d. Raw data (points), model predictions (lines), and the proportion of in situ temperature variability explained by SST data (adjusted *R^2^*) are shown for CoralTemp (blue), MUR (red), and MUR re-gridded at CoralTemp resolution (grey).


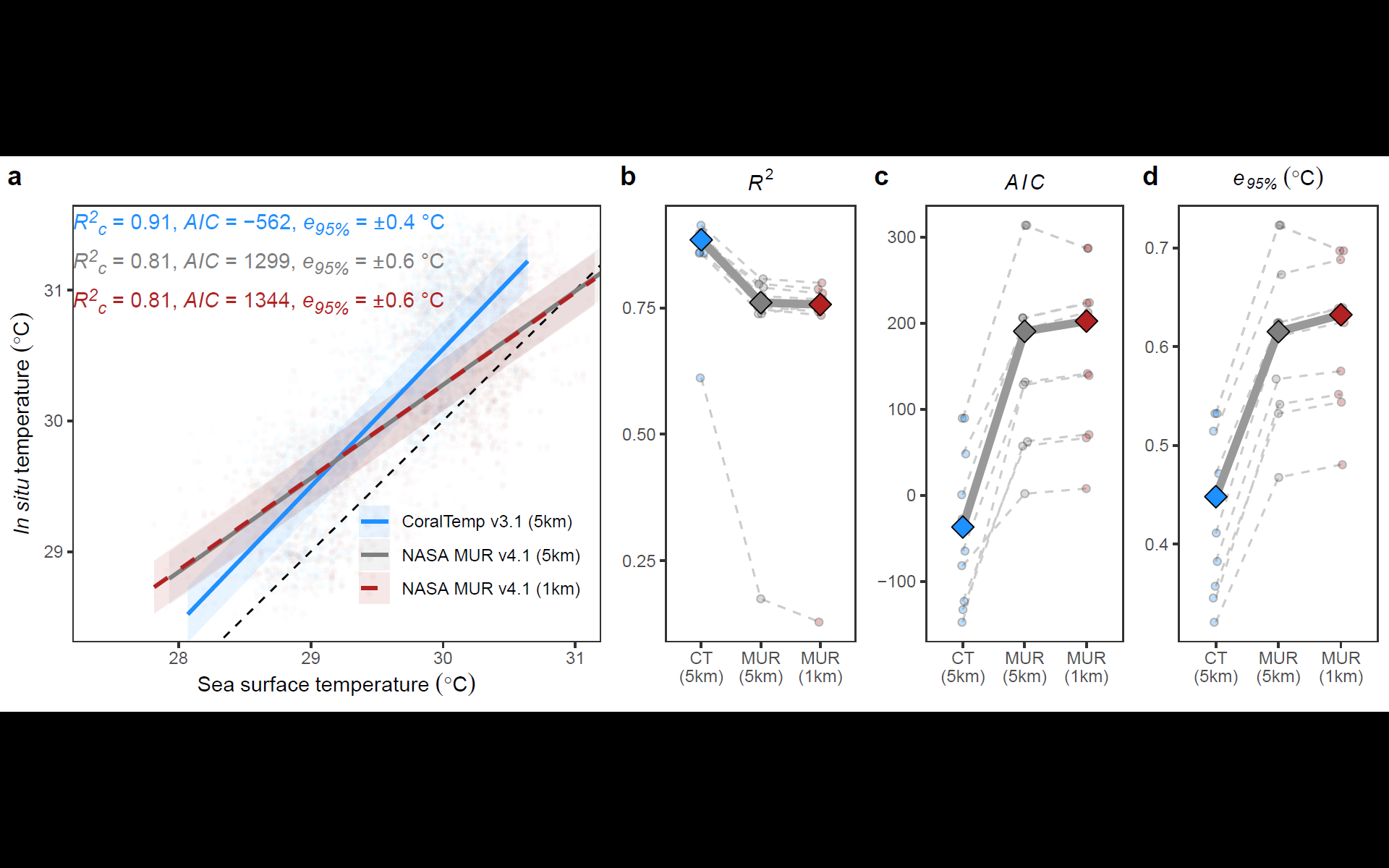


**Figure S4.** Reanalysis of SST ability to predict in situ logged temperatures across 11 deployed loggers at 5 sites for only the warm season (June – September). To be comparable to SST data, in situ data are first summarised as nightly averages. Panels descriptions the same as Fig. 2.


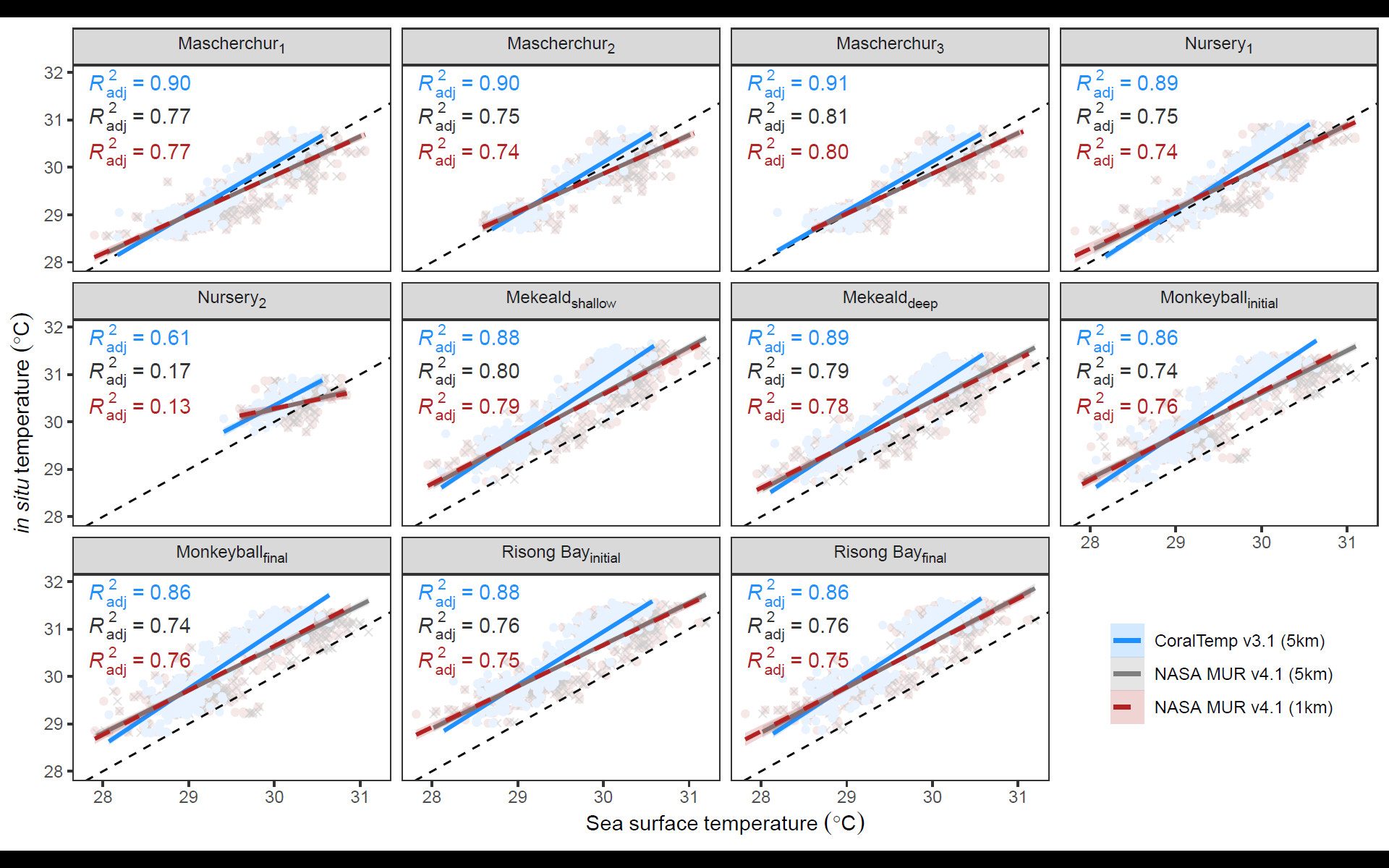


**Figure S5.** Reanalysis of site-specific linear regressions between sea surface temperature (SST) and nightly average in situ logged temperatures, but for only the warm season (June – September). Raw data (points), model predictions (lines), and the proportion of in situ temperature variability explained by SST data (adjusted *R^2^*) are shown for CoralTemp (blue), MUR (red), and MUR re-gridded at CoralTemp resolution (grey).


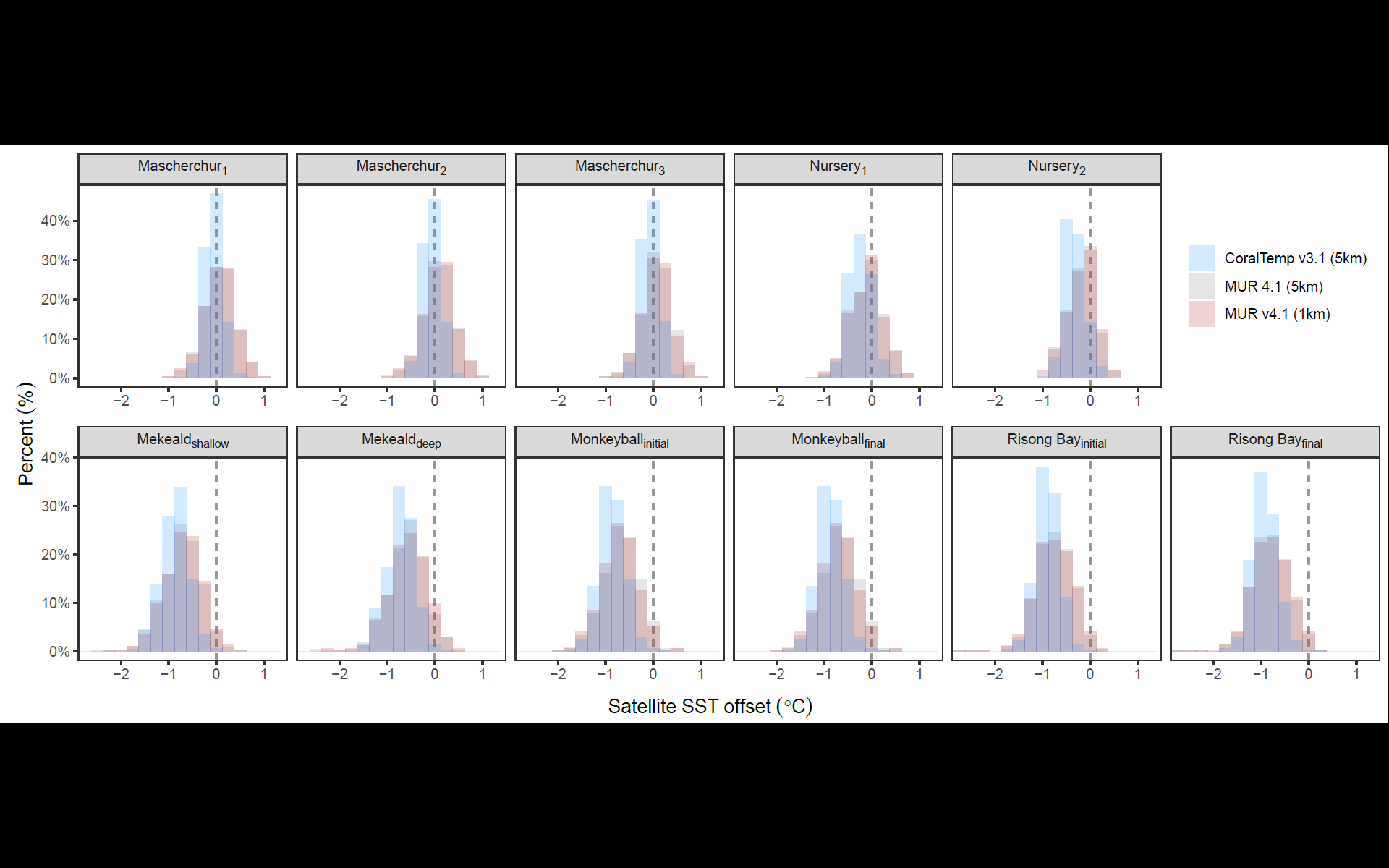


**Figure S6.** Histograms showing the accuracy and precision of SST data from CoralTemp (blue), MUR (red), and MUR re-gridded at CoralTemp resolution (grey), supporting Fig. 3.


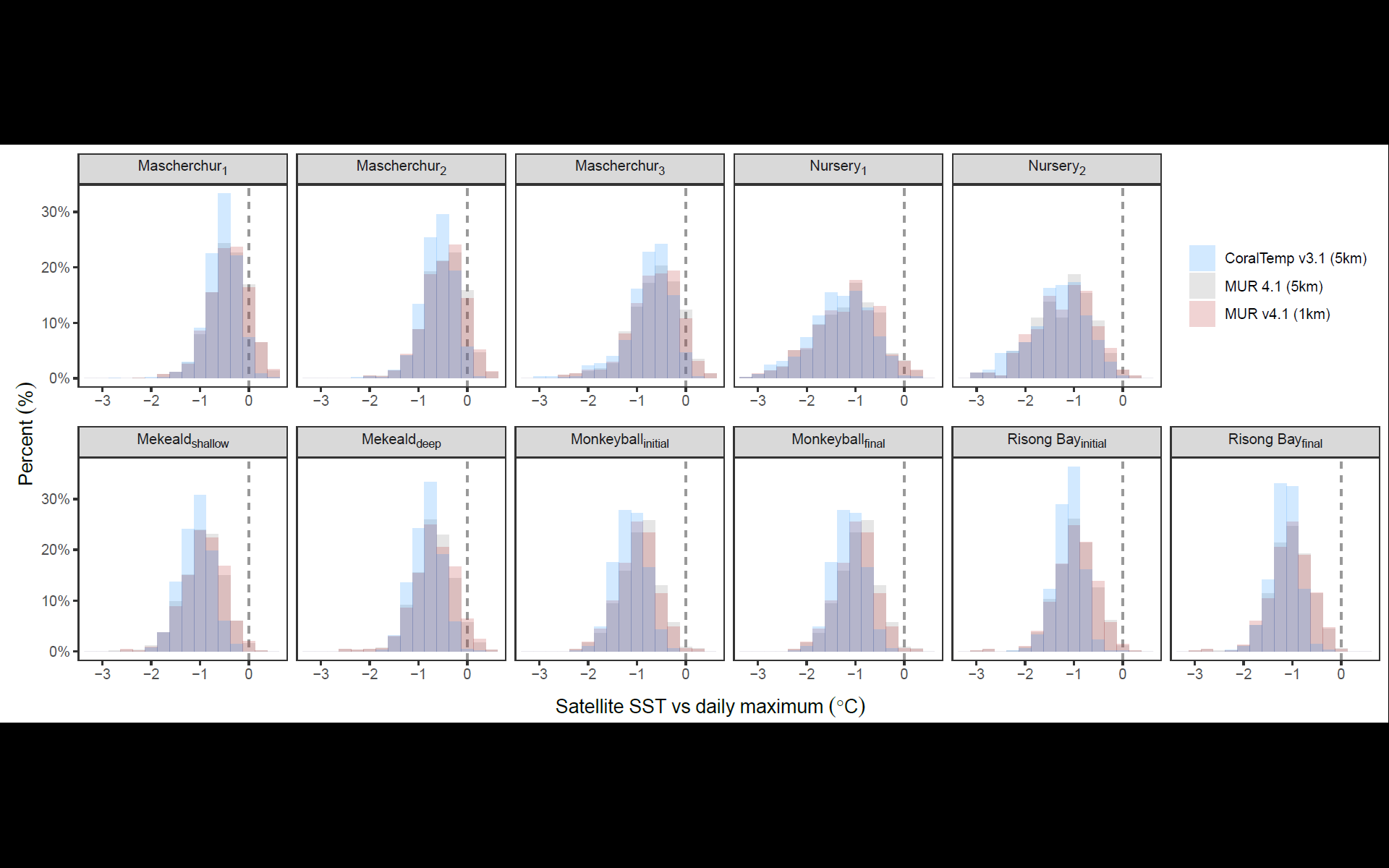


**Figure S7.** Histograms showing the difference between night-time SST data and in situ daily maximum from hourly logger data for CoralTemp (blue), MUR (red), and MUR re-gridded at CoralTemp resolution (grey), supporting Fig. 4.


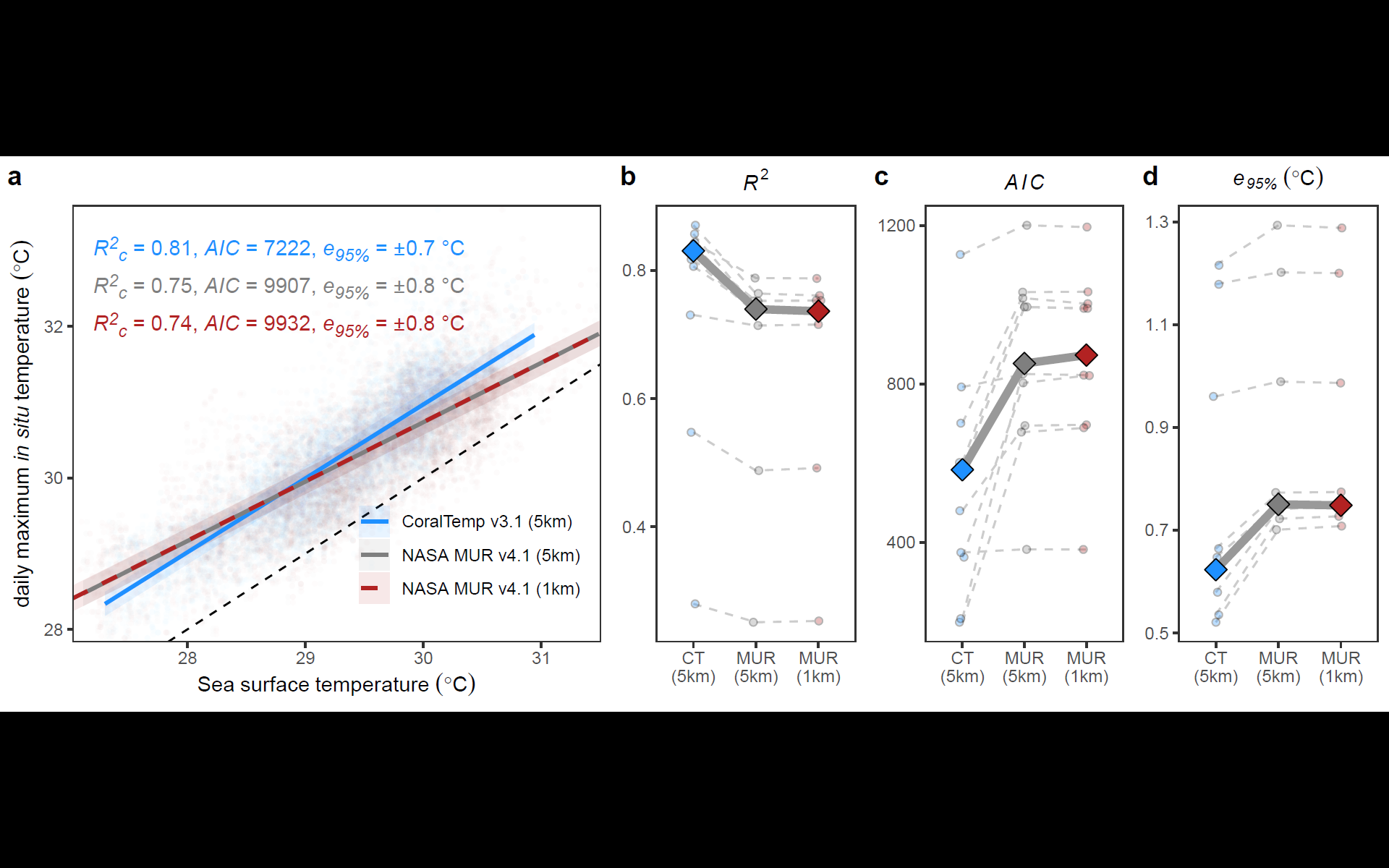


**Figure S8.** Ability of SST datasets to predict daily maximum in situ logged temperatures across 11 deployed loggers at 5 sites. (**a**) The overall relationship between daily maximum in situ temperatures (based on hourly data) and SST from CoralTemp (blue), MUR (red), and MUR re-gridded at CoralTemp resolution (grey), modelled with a linear mixed effects model, allowing for random intercepts for site and year. Model evaluation metrics show the proportion of in situ temperature variance explained by SST and random effects (conditional *R^2^*), overall goodness of fit (Akaike Information Criterion), and model error shown as a 95% error range (*e_95%_*, 1.96 × standard deviation of residuals). (**b**-**d**) The same summary statistics shown in (**a**) except based on linear regressions fitted for each site independently.

**Table S1.** Temperature logger positions and deployment times. Subscript numbers refer to location number with a site and subscript letters correspond to the following: s (shallow), d (deep), i (initial part of transect), and f (final part of transect).

| Site | Reef habitat | Longitude | Latitude | Depth (m) | Deployment start | Deployment end | Missing data (%) |
| --- | --- | --- | --- | --- | --- | --- | --- |
| Mascherchur_1_ | Outer | 134.5189 | 7.291679 | 2-3m | 12/11/2017 | 22/04/2022 | 0% |
| Mascherchur_2_ | Outer | 134.5197 | 7.291233 | 2-3m | 29/11/2017 | 15/01/2021 | 37% |
| Mascherchur_3_ | Outer | 134.5204 | 7.291106 | 2-3m | 30/11/2017 | 15/01/2021 | 39% |
| Nursery_1_ | Patch | 134.5019 | 7.3055 | 2-3m | 15/05/2019 | 31/03/2022 | 0% |
| Nursery_2_ | Patch | 134.5019 | 7.3055 | 2-3m | 11/06/2020 | 31/03/2022 | 0% |
| Mekeald_s_ | Lagoon | 134.4412 | 7.291456 | 2-3m | 19/11/2017 | 18/11/2020 | 0% |
| Mekeald_d_ | Lagoon | 134.4408 | 7.291264 | 9m | 05/06/2018 | 18/11/2020 | 0% |
| Risong Bay_i_ | Lagoon | 134.4841 | 7.306793 | 2-3m | 30/11/2017 | 19/11/2020 | 0% |
| Risong Bay_f_ | Lagoon | 134.4844 | 7.306465 | 2-3m | 09/09/2018 | 19/11/2020 | 0% |
| Monkeyball_i_ | Lagoon | 134.3719 | 7.230912 | 2-3m | 07/06/2018 | 18/11/2020 | 0% |
| Monkeyball_f_ | Lagoon | 134.3732 | 7.22968 | 2-3m | 28/11/2017 | 18/11/2020 | 0.04% |
